# Frying Oil Evaluation by a Portable Sensor Based on Dielectric Constant Measurement

**DOI:** 10.3390/s19245375

**Published:** 2019-12-05

**Authors:** Mei Liu, Xiangzheng Qin, Zhanghao Chen, Lei Tang, Brandon Borom, Ning Cao, Daniel Barnes, Kai Cheng, Jinbo Chen, Tao Wang, Jinjun Rao

**Affiliations:** 1 Shanghai Key Laboratory of Intelligent Manufacturing and Robotics, School of Mechatronic Engineering and Automation1, Shanghai University, Shanghai 200444, China; mliu@shu.edu.cn (M.L.); wdmzjqxz@163.com (X.Q.); wwzhuiwan@shu.edu.cn (Z.C.); 18717822095@163.com (L.T.); bborom@nevada.unr.edu (B.B.); flcshc@gmail.com (N.C.); danbarneso@nevada.unr.edu (D.B.); Caine_Cheng@shu.edu.cn (K.C.); chenjinbook@163.com (J.C.); wangt@shu.edu.cn (T.W.); 2 Department of Nutrition Science and Dietetics in the College of Agriculture, Biotechnology, and Natural Resources, University of Nevada, Reno, NV 89557, USA

**Keywords:** dielectric constant, interdigital electrode, total polar material, frying oil, capacitive sensor

## Abstract

A portable capacitive sensor was designed to assess frying oil degradation by measuring the changes in electrical capacitance. An interdigital electrode (IDE) was designed to be implemented as the testing probe (as IDEs are resistive to parasitic capacitance), together with an adjacent capacitive chip Pcap01 and a further microprocessor STM32, which were used as the data-processing elements. Experimental results demonstrated that viscosity could be a useful frying oil quality indicator, and also proved a preliminary correlation between IDE capacitance and oils’ total polar materials. This implies that IDE capacitance could be a suitable metric for conveniently assessing frying oil degradation. The designed capacitance sensor is light in weight, cost effective, and has excellent potential for simple, inexpensive, on-the-spot testing of the current quality of frying oil.

## 1. Introduction

A series of physical and chemical changes occurs in frying oil at high temperatures, including polymerization, oxidation and hydrolysis [1]. Aladedunye et al. studied the degradation and quality changes of the oil during the frying process and found that the polar components, acid value, and color change significantly with temperature [2]. Alireza et al. proved that with the extension of frying time, polymer content in frying oil increases, unsaturated fatty acid content decreases, the iodine value and saponification value decrease, and the acid value and peroxide value increase [3].

When testing the quality of frying oil, the measurement of total polar materials (TPM), provides the metric usually considered the most accurate since it includes critical information about the overall chemical degradation taking place in the oil. Column chromatography is the current standard method for measuring TPM. However, some of its drawbacks are that it is time consuming, requires a trained professional to operate, and requires many chemicals. Alternatively, researchers have since developed many new techniques to measure TPM-related parameters. For example, frying oil evaluation devices based on dielectric constant measurement are commercially available (e.g., CapSens 5000, AG., Wedensville, Switzerland; FOM 310, Ebro., Copenhagen, Germany; and Testo 270, Testo Inc., Baden-Württemberg, Germany). Through extensive use of these devices over time, a significant correlation between the dielectric constant and TPM in oil [4,5,6,7,8,9] has been established. These commercial devices have not been widely accepted, mostly because of their costly nature. 

Along with TPM measurements, researchers have developed other tools for measuring the quality of frying oil, which involve the relationship between the changes in polymer compounds and polar materials formed during frying. This relationship leads to increased viscosity and decreased interfacial tension, which are two factors that are considered useful indicators of frying oil quality in conjunction with TPM [10,11,12,13,14]. Other researchers have developed pore-based wicking sensors [15,16] and microfluidic methods [17,18], which function based on changing viscosity and interfacial tension and proved feasible for frying oil evaluation as their correlation with TPM is also quite satisfactory. 

Near-infrared spectroscopy (NIRS) is also a powerful tool in monitoring the quality of frying oil. It is capable of simultaneously measuring a number of chemical parameters, such as TPM, polymerized triglycerides (PTGs), free fatty acids (FFA), peroxides, anisidine value, and carbonyl value [19,20,21]. Other techniques attempted for frying oil assessment include image processing [22], E-nose [23,24,25], HPTLC-densitometry [26], and ultrasonic technique [27,28,29]. The main challenge for most of the equipment mentioned above is that they involve techniques that are bulky or expensive.

The primary goal of this project was to develop an easy-to-use, rapid, cost-effective, and portable device capable of assessing the quality of frying oil. The basic principle was to analyze the dielectric constant of frying oil via a capacitive sensor. Several research groups have attempted to develop capacitive sensors for frying oil using various smaller devices [30,31,32], but challenges included either the inevitability of parasitic capacitance [30] or the signal processing device being too bulky and expensive (i.e., LCR meter [31,32,33]). 

Our device is composed of three major components: an interdigital electrode (IDE) testing probe, an integrated capacitive chip, and a microprocessor. By tactful electric circuit and mechanical design, the whole structure is compact, light in weight, easy to integrate, and more resistive to external noise and parasitic capacitance. Preliminary results demonstrated a significant correlation between measured IDE electrical capacitance and TPM. Moreover, viscosity is also nearly linearly correlated with TPM. The designed sensor has great potential for simple and inexpensive field tests for frying oil quality. 

### 1.1. Principles

#### 1.1.1. Measuring Principles of TPM

As proven by previous researchers, frying oils’ dielectric constants are highly correlated with their TPMs [4,5,6,7], which means that by measuring the dielectric constant of the oil, the TPM can be obtained. 

**Figure 1:**
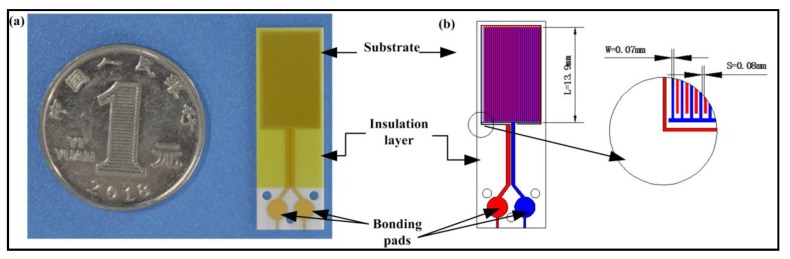
(**a**) A fabricated probe next to a 1 Yuan coin. The whole interdigital electrode (IDE) chip is 10 × 30 mm^2^; (**b**) A top view illustration of the designed capacitance sensor probe (electrode length L = 13.9 mm, electrode width W = 0.07 mm, electrode distance S = 0.08 mm, electrode pair number N = 28; bonding pad radius = 1.5 mm).

By ignoring peripheral effects, the electrical capacitance of the IDE in Figure 1 could be simplified and approximated as

(1) $$C\;=\;\frac{\varepsilon_{0}\varepsilon_{r}A}{S}\cdot N$$

where ε_0_ is the permittivity of free space constant, which is 8.854 pF/m; ε_r_ is the relative dielectric constant of the medium, i.e., frying oil in our case, which is around 2–4; S is the distance between electrodes; and N is the number of electrodes. A is the area of the electrode, which is calculated by the equation *A* = L W, where *L* and *W* are length and width of the electrode, as shown in Figure 1b. As shown in Equation (1), the capacitance C is proportional to the medium’s permittivity dialectric constant ε_r_, and it is also proportional to the specific oil’s TPM. By measuring the IDE capacitance C, the TPM can be obtained.

#### 1.1.2. Overall System Design

**Figure 2:**
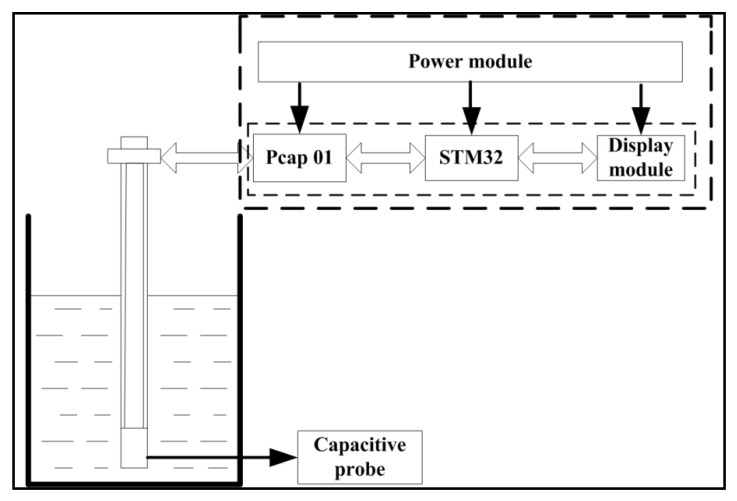
Working principles of the system.

A capacitor-based measurement system was designed, as shown in Figure 2, which used an IDE as the testing probe, a capacitor measurement chip Pcap01 (ACAM, Stutensee, Germany) as the signal transformation element, and an STM32 (STMicroelectronics, Geneva, Switzerland) as the signal processor and controller. The IDE itself was immersed in oil. As the dielectric constant of the frying oil changed, the capacitance of the IDE changed, according to Equation (1). The Pcap01 chip was used to achieve a precise measurement regarding the IDE capacitance and A/D conversion function and then transmitted the capacitive signal to the STM32 processor through serial peripheral interface (SPI) communication. The processor processed the capacitive signal and displayed the data on a dot-matrix screen. A power supply circuit carried out voltage conversion and provided power to the system.

## 2. Materials and Methods

### 2.1. Frying Oil Samples

**Figure 3:**
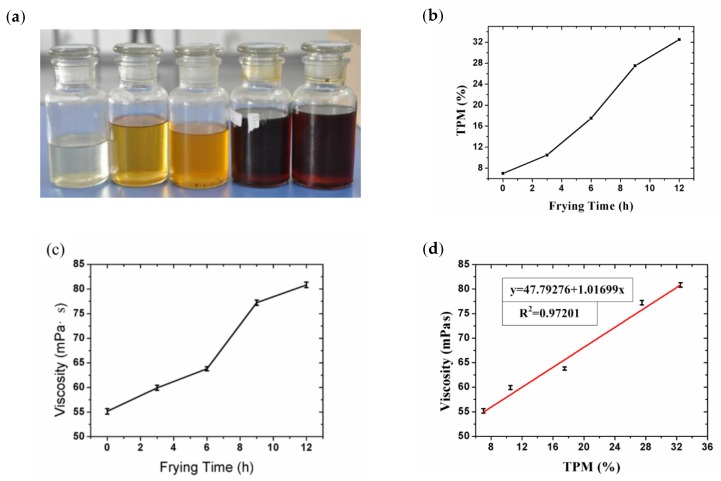
(**a**) Frying oil samples, with total polar materials (TPMs) ranging from 7–32.5%; (**b**) Frying oils’ TPM vs. frying time; (**c**) Frying oils’ viscosities vs. frying time; (**d**) Correlation between viscosities and TPMs at 21.8 °C.

The original oil (Fulinmen soybean oil) was purchased from Carrefour in Shanghai (French multinational retailer). The fatty acids in soybean oil are mainly unsaturated fatty acids. Before the experiment, soybean oil was produced and stored for three months. Initially, 1250 mL of oil was put in the frying pan and heated to a temperature of 200 °C. For each three-hour cycle, 100 mL of oil was removed from the pan to observe changes in the oil over time. During each three-hour cycle, six chicken legs (~0.5 kg) were cooked over a 1.5–2 h time period. After twelve hours of total frying, five frying oil samples were obtained, as shown in Figure 3a, including one original oil sample and four used oil samples. The TPMs of the frying oil samples were measured with a commercially available Testo 270 (Testo Inc., Baden-Württemberg, Germany) [8,17,34], and results are shown in Figure 3b. As viscosity was also a possible TPM indicator, oil viscosities were also measured with a rotary viscometer at room temperature (21.8 °C, NDJ-79; Shanghai Changji Geological Instrument Co., Shanghai, China), as shown in Figure 3c,d, providing different ways of evaluating our device’s accuracy.

### 2.2. Design and Fabrication of Interdigital Electrodes

The testing probe was one of the most important components in the device, as it was is in direct contact with hot frying oil and therefore needed to be very heat- and oil-resistive. The capacitive probe was designed based on the IDE shown in Figure 1. The sensor schematic was drawn using CAD software and fabricated with Ti/Cu/Ni/Au film on AlN (Aluminum Nitride) ceramic substrates by Guangzhou Hanji Sensing Scientific Company, LTD, China.

All other electrode fingers were electrically connected together through a common electrode arm. The electrode dimensions were designed to maximize the capacitance based on the maximum fabrication capability. The converging two electrodes at the bottom of the probe were designed to eliminate parasitic capacitance. Based on Equation (1), the capacitance C of the IDE in air is around 3 pF (ε_r_ is deemed as 1).

### 2.3. Signal Measurement System Design and Fabrication

#### 2.3.1. System Hardware Design

##### Working Principles of Capacitance Chip Pcap01

A key part of the entire sensor is the microcapacitance measurement circuit module. Its design is directly related to the accuracy measurement and anti-interference performance of the entire system. Pcap01 is a single-chip digital convertible capacitance chip with processing primarily used for capacitance measurement. Its advantages include high precision and low temperature drift. In addition, it can compensate for parasitic capacitance and can directly convert signals into digital mode with fast conversion speed. The chip connects the measured IDE capacitor (C) and the reference capacitor (C*_ref_*) to the same discharge resistor. The two capacitors are each discharged after being charged via the power supply voltage, and the discharge time is proportional to the value of the capacitor. The time-to-digital converter (TDC) works with high precision inside the chip and records the discharge time of both. In order to reduce the effect of temperature on the measurement results, the chip takes the discharge times of the two capacitors, that is, the ratio r = C/C*_ref_*, as the digital output [34,35,36,37].

The measurement resolution of Pcap01 is fixed, yet its measurement accuracy is affected by the reference capacitance value C*_ref_*. In addition, the C and C*_ref_* values should be in the same range in order to reduce the gain offset. To increase precision, a 56pF C0G chip type I ceramic capacitor with good temperature stability is selected as the reference capacitor C*_ref_*. To avoid parasitic capacitance interference, the design of the circuit should ensure that the trace of each capacitor input port is as short as possible. In addition, the capacitor to be tested is connected to the Pcap01 chip through the shielded line. For this reason, the IDE was directly screwed on the back of the electric circuit, through holes as shown in Figure 1, with the intention of eliminating parasitic capacitance.

**Figure 4:**
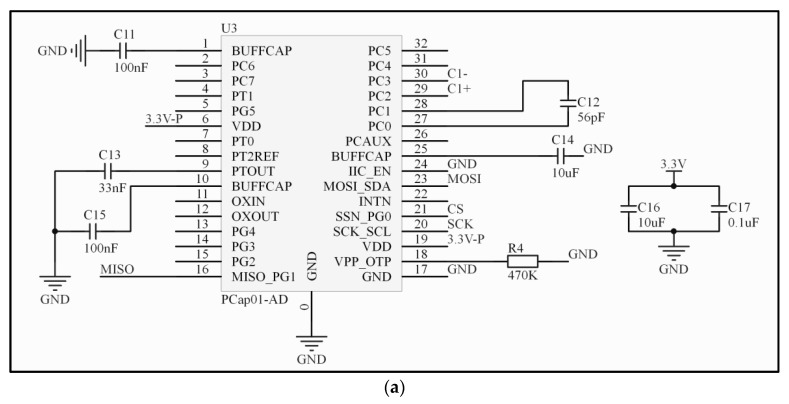

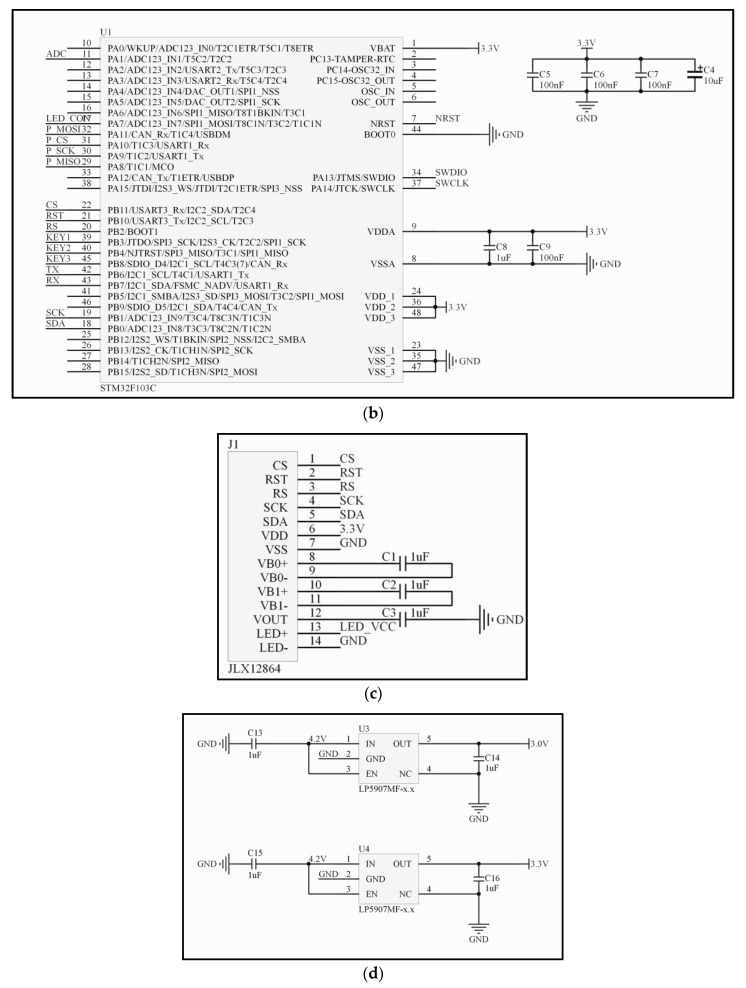
(**a**) Structure of the Pcap01 module; (**b**) Structure of the microprocessor STM32; (**c**) Circuit interconnection of the matrix screen; (**d**) LP5907 voltage regulator circuit.

Figure 4a shows that in the peripheral circuit diagram of the Pcap01 chip, only a small number of peripheral devices were needed, of which PC0 and PC1 were connected to the reference capacitor C*_ref_* and PC2 and PC3 to the IDE being tested. The chip communicated with the data processing module, i.e., the microcontroller, through SPI bus.

##### STM32 MCU Module, Display Module, and Power Management Module Design

The STM32F103C8T6 chip has several advantages: strong computing ability, low power consumption, and strong anti-interference ability. Its peripheral circuit is shown in Figure 4b. The MCU module reads the capacitance signal measured by Pcap01 and displays it on the connected screen.

In this paper, 128 × 64 dot-matrix screens of domestic 12864G-0088 were selected. Figure 4c shows the connection of each pin circuit, where the CS, RS, SCK, and SDA are connected to the IO ports of MCU, and the RST accesses a high level through a 1 kΩ resistance.

The working voltage of the main components of the test system is 3.3 V. The system is powered by a lithium battery with a voltage of 3.7–4.2 V, which is higher than the working voltage of 3.3 V, so a voltage reducing circuit should be used to reduce the voltage of the battery to 3.3 V. For the measurement of minute electrical capacity, the voltage must be stable without interference, so TI Company’s linear voltage-stabilizing chip TP4056 was selected, as shown in Figure 4d. The reduction efficiency was calculated as 3.3/3.7 × 100% = 89.2%, which met the low power consumption requirements dictated by the design. In addition, TP4056 was used in the charging management circuit.

#### 2.3.2. System Software Design

**Figure 5:**
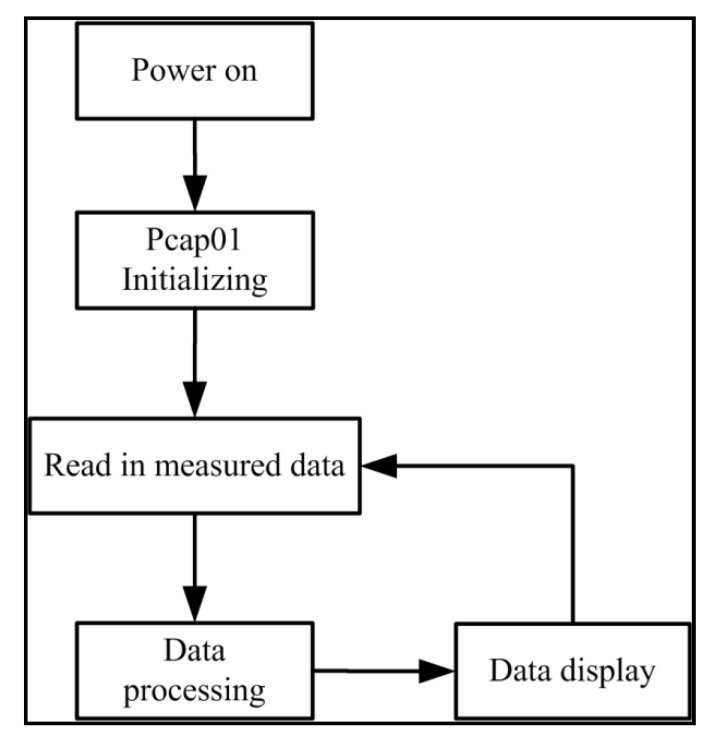
Capacitance measuring process.

This paper adopts the integrated development environment of IAR Assembler for ARM, using C language to write the development program of the microprocessor. This mainly includes the initialization of the Pcap01 chip and the collection, processing, and display of measured data, as shown in Figure 5.

As mentioned above, the output result r of Pcap01 is an unsigned fixed-point number with 3 integers and 21 decimals, which needs to be converted into a decimal. The final measured capacitance value C is

(2) $$C\;=\;C_{\mathrm{ref}}\frac{C_{2}}{2^{21}}$$

### 2.4. Device Installation and Test

**Figure 6:**
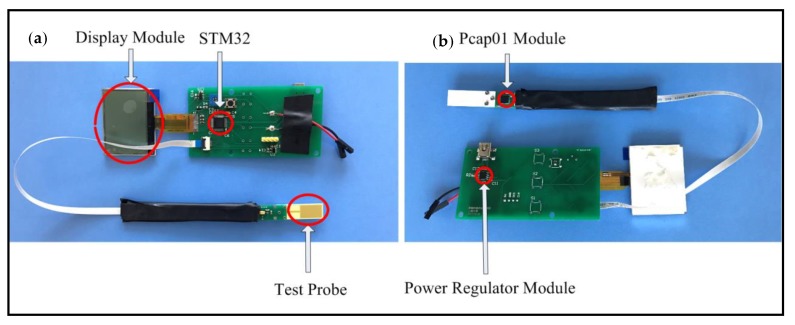
Sides of the fabricated capacitive device. (**a**) Front side; (**b**) Back side. Two series of tests were conducted, one of which was at room temperature and the other above 40 °C, the required working temperature of Testo 270. The testing IDE probe was immersed in frying oil samples over a period of 3 min, ample time to ensure accurate measurement. The data were then read and analyzed. Each measurement was conducted three times to ensure accuracy.

After the testing probe and electric circuits were completed, the whole device was constructed and tested. As shown in Figure 6, the IDE was as close to the Pcap01 chip as possible to eliminate parasitic capacitance. The length of the flexible flat cable was selected both for keeping the microprocessor away from oil and for easy operation. In our future plan, the PCB board holding the Pcap01 chip and the flexible flat cable would be encapsulated in a stainless steel tube, while the PCB board holding the microprocessor and power supply would be encased in a 3D-printed shell.

## 3. Results and Discussions

**Figure 7:**
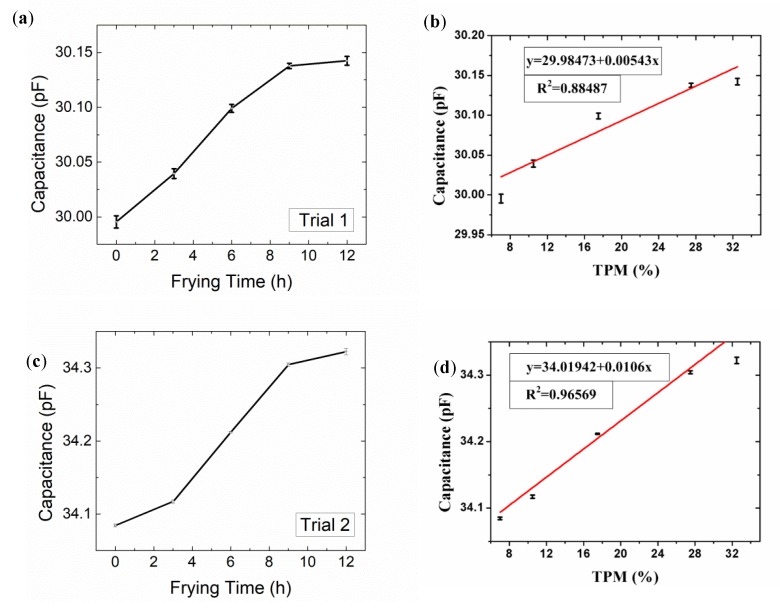
(**a**) Probe capacitance vs. frying time at 40–50 °C; (**b**) Correlation between probe capacitance and TPM (40–50 °C); (**c**) Probe capacitance vs. frying time at room temperature (25 °C); (**d**) Correlation between probe capacitance and TPM at room temperature (25 °C). *n* = 10.

As the frying time lengthened, the oil TPM appeared to increase consistently, as did its viscosity, as suggested in Figure 3, and the viscosity was also nearly linearly correlated with TPM, in accordance with the results of previous researchers [10,11,12,13,14]. The capacitance measurement data were also analyzed to evaluate the degradation of frying oil. Overall, the capacitance of frying oil increased as the heating time increased, as shown in Figure 7a,c. In our case, between 40 and 50 °C, the IDE capacitance rose from approximately 30.00 pF to 30.14 pF with heating time increasing from 0 to 12 h. Meanwhile, TPM increased from 7% to 32.5% with a resolution of 1.017% (compared to 0.5% of Testo 270). While the sample showed the same TPM at room temperature (25 °C), the capacitance rose from 34.08 pF to 34.32 pF as the device was more sensitive, and the resolution was better (0.298%, compared to 0.5% of Testo 270). The original capacitance of IDE in air at room temperature was 32 pF, on the same scale with the theoretical value 3 pF. The discrepancy is caused by moisture and simplification of the equation. The gap between capacitances at different temperatures indicates that as temperature increases, the dielectric constant of oil decreases. 

As shown in Figure 7b,d, the capacitance measured by the IDE sensor was highly correlated with TPM measured by Testo 270. In our case, the data measured at room temperature were most correlated with the results of Testo 270.

The fluctuation in the measured capacitance might be the result of static electricity and fluctuating moisture. The main drawback of this type of technique is that interference by moisture negatively affects sensor response; therefore, more extensive research in this area is recommended.

**Figure 8:**
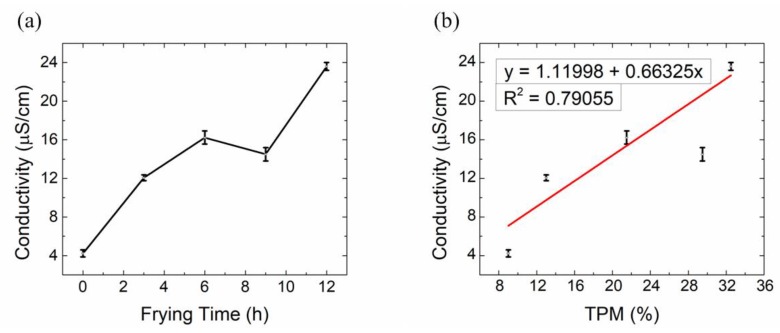
(**a**) Oil conductivity vs. frying time; (**b**) Correlation between oil conductivity and TPM. *n* = 3.

As there are contradictory conclusions on the conductivity of various frying oils (i.e., some research groups concluded that it is correlated with TPM and could be an oil quality indicator [38,39,40,41], while other groups concluded that it cannot [42,43]), this paper also tested the conductivity using a conductivity meter (senslONTM, Hach company, Loveland, Colorado, USA). As shown in Figure 8a, the conductivity generally increases with frying time; however, its correlation with TPM is not significant, as shown in Figure 8b, which means conductivity currently cannot be an indicator for oil degradation, in accordance with previous research [42,43,44]. 

Our results prove that viscosity could be a useful frying oil quality indicator, and they also represent a preliminary correlation between IDE capacitance and oils’ TPM, which implies that IDE capacitance could be a suitable metric for conveniently assessing frying oil degradation.

**Figure 9:**
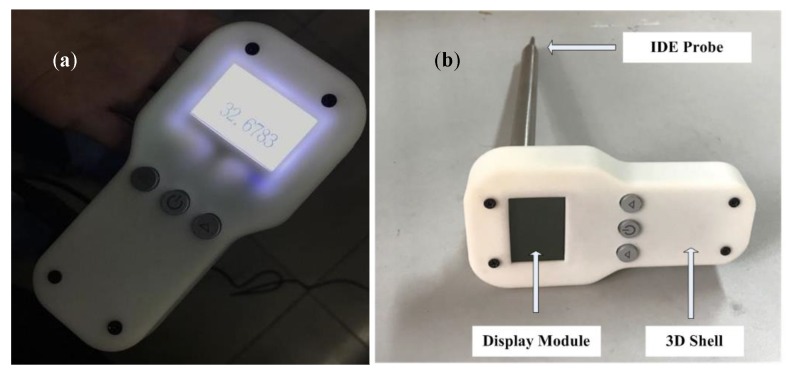
(**a**) On state of the frying oil evaluation device; (**b**) Off state of the frying oil evaluation device.

Several issues need to be addressed about the way to future field deployment. (1) A protective case could be 3D printed to better shield the microprocessor’s electric circuit. A tube should also be used to hold and encapsulate the Pcap01 chip and PCB circuit to insulate them from the oil more easily, as has been done for previous devices, as shown in Figure 9a,b. The tube could also hold the IDE on its tip. (2) Effects of static electricity should be considered and eliminated. (3) A temperature sensor could also be integrated. (4) As the sensor’s sensitivity changes with moisture/temperature, calibration should be taken into account. (5) Device reliability, precision, and accuracy should also be enhanced. (6) More samples should be tested to prove feasibility. 

## 4. Conclusions

The purpose of this project was to design an economical, portable, frying oil test device, which evaluated frying oil degradation based on dielectric constant. The device implemented an IDE as the testing probe and a Pcap01 and STM32 as the data acquisition and processing unit, which was simple, portable, and cost-effective. The preliminary testing result proved that the IDE capacitance is positively correlated with oils’ TPMs and can be a useful index for oil degradation. Viscosity is also an important index to measure the quality of frying oil, which is nearly linearly correlated with TPM. The next step is to improve the reliability and steadiness of the device by adding a temperature sensor and optimizing electric circuit design, capsulation, packaging, etc.
